# Sex, Gender Identity, and Perceived Employability Among Spanish Employed and Unemployed Youngsters

**DOI:** 10.3389/fpsyg.2018.02467

**Published:** 2018-12-07

**Authors:** Eva Cifre, María Vera, Israel Sánchez-Cardona, Nele de Cuyper

**Affiliations:** ^1^Research Group Género, Salud y Trabajo, Department of Developmental and Social Psychology and Methodology, Universitat Jaume I, Castellón, Spain; ^2^Department of Education and Social Psychology, Universidad Pablo de Olavide, Seville, Spain; ^3^PhD Clinical Psychology Program, Carlos Albizu University, San Juan, Puerto Rico; ^4^Research Group Work, Organizational and Personnel Psychology, Faculty of Psychology and Educational Sciences, KU Leuven, Leuven, Belgium

**Keywords:** qualitative and quantitative, perceived employability, gender identity, sex, employment, unemployment, youngsters

## Abstract

Young people find it difficult to access to the labor market, particularly in countries like Spain with a dramatically high rate of unemployment. A further problem is that this labor market is not gender-neutral. This has been demonstrated repeatedly in the literature, with women typically being at a disadvantage. This highlights the need to study issues related to employability from a gender perspective, beyond including sex as a mere control variable. This analysis is relevant given the gender biases in organizations and in society in general that hinder the advancement of gender equality in organizations. Accordingly, our aim is to study both sex (male vs. female) and four profiles of gender identity based on dimensions of masculinity and femininity (i.e., feminine, masculine, undifferentiated, and androgynous) in relation to perceived employability in an exploratory way in two samples of employed (*N* = 181) and unemployed (*N* = 246) Spanish youngsters (i.e., below 30). The results show different patterns for employed and unemployed youngsters regarding sex, gender identity and their interaction in relation to perceptions of being employable. Concerning sex, women seem more confident about their employment chances when unemployed. In contrast, men feel more confident about their employment chances within their organization than women when employed. Concerning gender identity, the androgynous gender profile in the employed sample (in both men and women) scored highest on perceived employability. Results of the sex–gender identity interaction show that being feminine associates with the highest level of perceived employability for an unemployed man and the lowest for an unemployed woman. Moreover, both unemployed men and women androgynous score the highest in perceiving employability (except feminine men). Our findings highlight that sex and gender identity do play a role in shaping employability perceptions of young men and women in different labor contexts (employment and unemployment). This reinforces the need of changes against discrimination at work and in job search from a feminist approach to arrive at a more equal society.

## Introduction

The labor market and employment are gendered in multiple ways. For instance, young men under 29 in Spain are more likely to be unemployed (9,73% men and 8,41% women) and women are more likely to work part-time (7,3% men and 24,2% women) (Active Population Survey, by the Instituto Nacional de Estadística [INE], 2018). Additionally, segregation still exists across jobs and sectors (i.e., horizontal segregation) and in hierarchal structures of the organizations (i.e., vertical segregation) (i.e., Hakim, 1993; Huffman et al., 2010; Baird, 2012; Organization for Economic Co-operation and Development [OECD], 2018).

Despite these findings, gender has not yet attracted much attention in employability research. Employability concerns the individual’s chance in the internal and/or external labor market (Forrier et al., 2009) and is advanced as critical research area to achieve sustainable working lives (De Vos et al., 2018). Employability studies have primarily included gender as a control variable (e.g., Kalyal et al., 2010; De Cuyper and De Witte, 2011; González-Romá et al., 2018) but from a fairly narrow perspective, namely in terms of sex and without any gender interpretation. There are some hints to the role of gender in the conceptual and theoretical debate about employability (e.g., Fugate et al., 2004) and work related studies (Kanfer et al., 2001; Smith et al., 2005), yet empirical studies are lagging behind. More specifically, gender identity, or the degree in which the person assumes gender roles as part of their identity, has not been considered in employability research. While sex may determine how individuals are viewed by labor market actors, assumed gender roles are more likely to be stronger predictors of how individuals approach and behave in relation to the labor market.

Accordingly, our aim is to examine the relationship between gender and employability. We approach gender broadly in terms of both sex and gender identity. Sex and gender are sometimes used interchangeably, although both refer to conceptually distinct attributes. According to the World Health Organization [WHO] (2011), sex refers to the different biological and physiological characteristics of males and females, such as the reproductive system, chromosomes, hormones, etc. Gender refers to the socially constructed characteristics of women and men such as norms, roles and relationships of and between groups of women and men. To simplify, in this study, we will use the word “sex” to consider these sex/gender differences, and “gender identity” as the assumed roles derived of that sex distinction.^1^ We conceive employability as the individual’s appraisal of his/her chance in labor market, coined perceived employability. Those appraisals may concern the internal (i.e., within the current organization) or the external (i.e., with another employer) labor market and jobs in general or better jobs. We focus upon appraisals, as they are the main drivers for career behavior (Van Hercke et al., 2014; Forrier et al., 2015). In particular, if men and women perceive different chances in the labor market, they will behave accordingly, and this may contribute to gender segregation.

A strong feature of this study is that we explore differences in perceived employability based on sex and gender identity in a sample of employed and unemployed individuals in Spain younger than 30. Those samples are particularly well suited for a number of reasons. First, young adults have grown-up in a more gender equal society and therefore they may not follow the traditional gender roles or incorporate those roles to their identity. Hence, it would be interesting to examine if and how sex and gender identity affects employability. Second, employability has particular resonance among young adults who enter the labor market, particularly in Spain: The situation in Spain is certainly worrisome, with general unemployment above 17% (Instituto Nacional de Estadística [INE], 2018 second trimester) and the unemployment among young adults double this percentage (36.3%, Active Population Survey, first semester 2018). Youngsters who have obtained a job may experience high levels of job insecurity due to the instability of their jobs (i.e., Peiró et al., 2007). Perceived employability has particular resonance in this context since it reduces job insecurity (De Cuyper et al., 2012), buffers the negative consequences associated with job insecurity (e.g., Silla et al., 2009; Kalyal et al., 2010; Green, 2011) and promotes job search (Koen et al., 2013), and well-being (De Cuyper et al., 2017) among the unemployed. Third, the two different samples will help to cross-validate results across different labor market conditions, namely employment and unemployment.

### Sex, Gender, and Perceived Employability

As human beings are embedded into a culture, it is difficult to disentangle sex and gender in colloquial language and scientific writing (Wood and Eagly, 2015). Sex often connotes sexuality, while gender consists of the meanings ascribed to male and female social categories within a culture. However, in a practical way, sex and gender are generally difficult to distinguish. For example, we do not know whether our choices and behaviors are due to only biological variables (sex) or their interaction with our society norms (gender). Thus, it is challenging to differentiate male-female (sex) from men-women (gender).

Sexual differences are framed by gender roles, which are strongly enchained in society, and refers to the shared beliefs that apply to individuals on the basis of their socially identified sex (Eagly, 1987; Eagly et al., 2004). Men and women tend to specialize in different behaviors in most cultures, and this has led to different beliefs about what men and women can and should do (Wood and Eagly, 2010). Those beliefs are often described along two dimensions: agency and communion. Men are assumed to be agentic, typically described as being masterful, assertive, competitive and dominant. Conversely, women are assumed to be communal along descriptions such as friendly, unselfish, concerned with others and emotionally expressive. Thus, jobs that require those agentic or communal characteristics will be perceived as more suitable for men or for women, respectively. Also those gender roles will foster women’s and men’s interests toward not only different kind of jobs (horizontal segregation), but also toward which level they might aspire to assume (vertical segregation) and even how important should be working on the public sphere.

According to the Social Role Theory (Eagly, 1987), men would stereotypically be more oriented to work in the public sphere (breadwinner) and being encouraged by society to take up this role. Traditionally, it is expected that men would show agentic traits that will allow them to work in the public sphere providing support to their family. By comparison, women would stereotypically be more oriented to work in the private sphere (care-taker). Based on this traditional division of labor, it is expected that women would show communal traits and be oriented toward occupations related to care taking (i.e., nursering, teaching) and less orientation toward working in the public sphere, and when doing so, working in jobs compatible with their private sphere “responsibilities.”

Socially embedded gender roles may affect employment chances in two ways, obviously interrelated. First, employers may have views on what are gender-appropriate jobs, and may recruit and hire accordingly. This affects the labor market chances of men and women differently. For example, Heilman (2012) analyzed how gender stereotypes promote gender bias in the workplace, and this hampers women promotion. Second, gender roles may influence the way men and women themselves approach the labor market and the available employment choices. Assigned gender roles may influence first career choice and then the perception of competence or success in getting and maintaining a job (Correll, 2001). This idea has attracted some attention from career scholars, for example along insights from Social Cognitive Career Theory (for an illustration, see Williams and Subich, 2006). Common to both perspectives is the idea that certain jobs are “for men” (masculinized jobs) or “for women” (feminized jobs); or that only one a person from a specific sex (e.g., male) might have more chances to get to the top level of organizations (e.g., managers). Therefore, sexual differences might affect employability of women and men.

Taking this one step further, sex differences may likewise affect perceived employability. Perceived employability concerns chances a person sees in the internal (i.e., perceived internal employability, with the current employer) and the external labor market (i.e., perceived external employability; with another employer). The distinction between internal and external employability is generally accepted (Rothwell and Arnold, 2007): the underlying idea is that perceived employability is shaped through the interaction between personal and contextual features, and the distinction between perceived internal and external employability accounts for different contextual dynamics. For example, perceptions of internal employability may in part be shaped through Human Resources practices and internal promotion opportunities and perceptions of external employability through the general economic climate (Van Hercke et al., 2014). Chances in the labor market may refer to *any* job (i.e., perceived quantitative employability) or better jobs (i.e., qualitative employability). The focus upon quantity vs. quality is relatively new and unexplored, though first results are promising (see e.g., De Cuyper and De Witte, 2011). The underlying idea here is that the perceived quality of job opportunities is a signal of employee’s worth in the labor market and a stronger urge for career progression. The combination leads to four types of perceived employability: perceived internal quantitative employability, perceived internal qualitative employability, perceived external quantitative employability and perceived external qualitative employability. The four types can be meaningfully distinguished and related differently to work behaviors. Then, although we might expect that men would have higher perceived employability than women in general, it seems interesting to explore if this holds across the different aspects of perceived employability. A plausible assumption is that gender differences are particularly salient in the external labor market, when employers do not have person-specific information and hence rely more easily on general stereotypes. Another assumption could be that gender differences are larger when quality indicators are accounted for.

Based on these argument, we propose the following research question to further explore the influence of sex and gender on perceived employability:

RQ1: Do men and women differ in perceived internal quantitative, perceived internal qualitative, perceived external quantitative and perceived external qualitative employability?

### Gender Identity and Perceived Employability

When people incorporate the cultural meanings of what it is to be a man or a woman into their own psyches, gender becomes part of their identities (Wood and Eagly, 2015). Through these gender identities, individuals may think and act according to these gendered aspects of their selves (Wood and Eagly, 2010), including their approach to the labor market.

Thus, gender identity may likewise affect perceived employability. A plausible assumption is that individuals who adopt a masculine identity might be more oriented toward working in the public sphere and thus more actively seek out employment opportunities than those with a feminine identity, who might more oriented to the private sphere. Also, there are certain characteristics that are regarded as masculine who seems be desirable to certain work/employment domains (i.e., competitiveness, leadership). Accordingly, more masculine individuals (men or women) might feel a higher perceived employability than the feminine ones.

Yet, this picture becomes much more complicated when considering that gender identity is fluid rather than binary. Said differently, gender identity based on gender-stereotypical personality traits should not be conceived as a strict divide between men-masculine and women-feminine. Instead, individuals might score high on both masculinity and feminity. This has led to four gender identity profiles that are commonly accepted in the literature (e.g., Bem, 1974): feminine (high on feminity, low on masculinity), masculine (low of feminity, high on masculinity), undifferentiated (low on both feminity and masculinity) and androgenous (high on both feminity and masculinity). Some studies (i.e., Gartzia et al., 2012) found that individuals with an androgenous profile show higher emotional intelligence, which leads to a better adaptation to different situations. This is because they might show the masculine or the feminine traits contingently as required by the situation. In this sense, we might expect these people will perceive to be more employable as they might adapt better to different jobs profiles. Our point here is that gender identity might play a role, though which role exactly and how the different gender identity profiles compare to each other in relation to perceived employability is unclear. Accordingly, our second research question is as follows:

RQ2: Do individuals with different gender identity profiles differ in perceived internal quantitative, perceived internal qualitative, perceived external quantitative and perceived external qualitative employability?

### Sex, Gender Identity, and Perceived Employability

As we stated before, employability research has considered gender interchangeably with sex as a covariate or predictor and has not extensively studied the role of gender identity. We extend this by examining the combined effect of sex and gender identity in perceived employability, which portraits a more complex scenario. Only few studies have analyzed the combined effects of sex and gender identity on psychosocial variables at work (i.e., on perceived stress by students; Jones et al., 2016), although none of them in actual occupational settings. With respect to perceived employability, a plausible assumption is that women who strongly identify with the female identity may be less oriented toward the labor market than men who strongly identify with the male identity who actively seek and pursue more job opportunities. However, the question is how men and women with gender identity profiles different to the ones that traditionally would be assigned based on their sex (i.e., non-masculine men, non-feminine women) appraise their employability. For example, it has been found that women working in STEM jobs need to reduce their feminity expression to better integrate in this stereotypically masculine jobs (i.e., Eisenhart and Finkel, 1998; Faulkner, 2000). Additionally, studies in the police force, as a male-dominated field has found that gender-dissimilarity in police teams was related to perceived gender-work identity conflict for women, but not for men (Veldman et al., 2017). Men might also be affected by non-congruity between their sex and gender identity. It is known that masculine identification correlates with positive attitudes toward male and female gender identity types that conform to traditional gender norms (i.e., masculine men, feminine women), but negative attitudes toward feminine men (Glick et al., 2015), which might affect young men looking for or maintaining a stereotyped feminine job. The following research questions address these issues:

RQ3: How do sex and gender identity interact in relation to perceived internal quantitative, perceived internal qualitative, perceived external quantitative and perceived external qualitative employability?

## Materials and Methods

### Sample and Procedure

#### Sample 1: Employed

Data were collected by a consultant group specialized in human resources, and hired for this specific purpose. The consultant group approached their clients’ employees with an invitation to participate in the survey during June and October 2015. Invitations were sent to members under 30 years with at least 2 years of work experience employed in 20 production, retail and service organizations. Questionnaires were completed online and through paper-and-pencil. Participation was voluntary and confidential. Respondents were provided with an individual feedback report about psychosocial risks factors at work as token of appreciation.

The final sample for this study consisted of 181 employees. About half of the sample was male (47% male and 53% female), and mean age was 26.1 (*SD* = 3.2). Regarding education, 1.1% did not obtain a degree, 8.4% completed basic education, 17.3% professional education, 8.4% high school, 40.2% university, and 24.6% postgraduate studies. Regarding occupational position, 13.6% were managers, 35.2% qualified staff, and 51.2% had auxiliary or apprentice jobs.

#### Sample 2: Unemployed

The researchers contacted the public employment service of the Valencian Community (Spain). This employment service invited individuals younger than 30 years who were unemployed for at least 6 months at the moment of participation (but who had had at least 2 years of work experience) and available for work and searching for a job to a compulsory activity as part of their reemployment plan. Once there, the researchers asked them to participate in the research project. They were asked to complete the questionnaire, voluntarily and confidentially. The data collection was exclusively in paper-and-pencil. In exchange for their collaboration, researchers provided participants with a free training course about the use of emotional regulation during their search for a new job.

This sample included 237 unemployed people. About half of the sample was male (52% male and 48% female) with a mean age of 26.7 (*SD* = 2,4). Regarding education, 10.2% did not obtain a degree, 40% basic education, 23.4% professional education, 14% high school, 7.7% university studies, and 4.7% had postgraduate studies. Finally, participants have been, on average, 22.9 months unemployed (*SD* = 19.02).

### Instruments

*Sex:* Participants were asked to indicate their identified gender: man (0) or woman (1).

*Gender identity:* Participants completed the short 12-items Spanish version of Bem Sex-Role Inventory (BSRI, Bem, 1974), validated by Mateo and Fernández (1991). This is a measure of gender expression that includes six items of masculinity, (e.g., dominant) and six items of femininity (e.g., kind). Participants answered using a 7-point Likert scale ranging from 1 (never) to 7 (always). Following Carver et al. (2013) and Vafaei et al. (2014), we used the median split method to compute the four gender identity profiles. Firstly, we calculated the median for the masculine and feminine scales. Secondly, individual scores for each participant on the femininity scale and the masculinity scale were calculated and compared to the median. Thirdly, groups were created following this rules: (1) if the participant’s mean scores on both the masculine and feminine scales were equal to or above the median that participant was classified as androgynous; (2) if the participant mean score was below the median on both the feminine and masculine scales, the participant was classified as undifferentiated; (3) those participants whose mean score were equal to or higher than the median on the masculine scale and lower on the feminine scale were classified as masculine; (4) those participants who were equal to or higher than the median on the feminine scale and lower on the masculine scale were classified as feminine. In the Spanish version of the BSRI-12, Mateo and Fernández (1991) found the coefficients of internal consistency ranged from 0.83 to 0.94.

*Perceived Employability* was measured with the Spanish version of the scale developed by De Cuyper and De Witte (2010) and De Cuyper and De Witte (2011) in *Sample 1* (employed). We used four dimensions, with four items each: (1) *Internal quantitative* (e.g., I am optimistic that I could find another job, if I looked for one); (2) *Internal qualitative*, (e.g., I am optimistic that I could have a better position within the company); (3) *External quantitative* (e.g., It would be very easy to get a similar job in another company); and (4) *External qualitative* (e.g., I am optimistic that I could find a better job elsewhere, if I looked for one). The original studies report good internal consistency with the following reliabilities (Cronbach’s a): 0.91, 0.94, 0.95, and 0.96, respectively. In *Sample 2* (unemployed), we adapted the scale described above to fit the situation of the unemployed individuals. In particular, we focused upon the external labor market. Thus, perceived internal employability was not considered and included in this version of the scale as it has no meaning for unemployed individuals. Moreover, we changed the four items for *External quantitative* (e.g., I can easily find a job.) and the four items for *External qualitative*, (e.g., I can easily find a better job than I had previously) from “find another job” to “find a job.” In all cases, participant answered on a 7-point Likert scale from 0 (strongly disagree) to 6 (strongly agree).

### Data Analysis

We performed three steps to analyze the data. First, we ran descriptive analyses with specific attention to gender and sex frequency distributions in both samples. Second, confirmatory factor analyses were performed to validate the four (Sample 1) and the two (Sample 2) dimensions of the perceived employability scale. The goodness-of-fit of the models was evaluated using absolute and relative indexes. The three absolute goodness-of-fit indexes calculated were: (1) the χ^2^ goodness-of-fit statistic; (2) the Goodness-of-Fit Index (GFI); and (3) the Root Mean Square Error of Approximation (RMSEA). Additionally, we computed a relative index: Comparative Fit Index (CFI). Because the distribution of the GFI is unknown, no statistical test or critical value is available (Jöreskog and Sörbom, 1993). Values below 0.06 for the RMSEA are indicative of an acceptable fit (Hu and Bentler, 1999), whereas a cut-off value close to 0.95 for CFI is considered to indicate an adequate model fit (Hu and Bentler, 1999). Confirmatory factor analyses were performed with AMOS 21. Third, multivariate analysis of variance (MANOVA) were performed to test sex, gender and their interaction effects on dimensions of perceived employability. *Post hoc* analyses through Bonferroni test were performed to further analyze any significant differences. Multivariate analyses were performed with SPSS 22.

## Results

### Descriptive Analysis

Table 1 shows means, standard deviations, alphas and correlations for each dimension of perceived employability in both samples. Qualitative perceived employability had lower means than quantitative perceived employability for both perceived internal and external employability and across samples. Correlations and reliability estimates (Cronbach’s alpha) were as expected. Cronbach’s alphas for gender identity were 0.80 for employee and 0.75 for unemployed.

**Table 1:** Means, standard deviations, and correlations for employed (*n* = 182) and unemployed (*n* = 237).

	**Employed**			**Unemployed**			1	2	3	4
			
	*M*	*SD*	*α*	*M*	*SD*	*α*				

1. External quantitative	3.02	1.42	0.86	2.38	1.28	0.81	1	0.76^∗∗∗^	0.21^∗∗^	0.28^∗∗∗^
2. External qualitative	2.63	1.29	0.86	2.10	1.24	0.79	0.88^∗∗∗^	1	0.25^∗∗∗^	0.35^∗∗∗^
3. Internal quantitative	3.05	1.36	0.76						1	0.74^∗∗∗^
4. Internal qualitative	2.35	1.28	0.79							1

*^∗∗∗^*p* < 0.001; ^∗∗^*p* < 0.01; S_*1*_, Sample 1 (employed); S_*2*_, Sample 2 (unemployed); under diagonal correlations of S_*1*_, above diagonal correlations of S_*2*_*.

In order to descriptively visualize the patterns of variability on perceived employability, Tables 1, 2 show the means, and standard deviations for perceived employability among the samples of employed (Table 2) and unemployed (Table 3) taking into account sex and gender identity: men and women (sex) and, within this, androgynous, undifferentiated, masculine and feminine (gender identity). Within each of these eight groups, means and standard deviations are shown.

**Table 2:** Descriptive statistics of employability by sex and gender identity among employees (*n* = 182).

	**Men (*n* = 85)**								**Women (*n* = 97)**							
	1 (*n* = 25)		2 (*n* = 20)		3 (*n* = 32)		4 (*n* = 8)		1 (*n* = 30)		2 (*n* = 17)		3 (*n* = 24)		4 (*n* = 27)	
	*M*	*SD*	*M*	*SD*	*M*	*SD*	*M*	*SD*	*M*	*SD*	*M*	*SD*	*M*	*SD*	*M*	*SD*

External quantitative	3.43	1.37	2.81	1.33	2.79	1.07	3.03	1.88	3.21	1.60	2.53	1.40	3.31	1.47	2.90	1.47
External qualitative	3.16	1.28	2.43	0.98	2.36	1.16	2.44	1.76	2.93	1.51	2.13	1.08	2.59	1.38	2.68	1.15
Internal quantitative	3.78	1.40	3.02	0.99	3.20	1.22	3.13	0.95	2.98	1.56	2.63	1.49	3.08	1.51	2.47	1.11
Internal qualitative	2.96	1.38	2.43	0.94	2.60	1.18	2.38	1.08	2.44	1.51	1.81	1.01	1.96	1.44	2.02	1.09

*1 = androgynous; 2 = undifferentiated; 3 = masculine; 4 = feminine*.

**Table 3:** Descriptive statistics of employability by sex and gender identity among unemployed (*n* = 237).

	**Men (*n* = 124)**								**Women (*n* = 113)**							
	1 (*n* = 27)		2 (*n* = 37)		3 (*n* = 34)		4 (*n* = 26)		1 (*n* = 34)		2 (*n* = 22)		3 (*n* = 7)		4 (*n* = 50)	
	*M*	*SD*	*M*	*SD*	*M*	*SD*	*M*	*SD*	*M*	*SD*	*M*	*SD*	*M*	*SD*	*M*	*SD*

External quantitative	2.52	1.42	1.78	1.09	2.34	1.60	2.52	0.87	2.71	1.34	2.59	1.22	3.32	0.69	2.27	1.17
External qualitative	2.27	1.44	1.32	1.03	1.88	1.53	2.34	0.95	2.32	1.24	2.48	1.17	2.96	0.85	2.16	1.04

*1 = androgynous; 2 = undifferentiated; 3 = masculine; 4 = feminine*.

Results in Table 2 show that both employed men and women with an androgynous profile felt more employable on all the dimensions. The pattern was different in the unemployed sample (Table 3) feminine men and masculine women reported the highest levels of external qualitative and quantitative perceived employability. Note also that there are more feminine men and women in the unemployed sample compared to the employed one (*n* = 8 feminine men in the employed sample vs. *n* = 26 in the unemployed one; *n* = 27 feminine women in the employed sample vs. *n* = 50 in the unemployed one). The number of masculine unemployed women is the lowest (*n* = 7).

### CFA

Confirmatory factor analyses were performed to test the four (sample 1) and the two (sample 2) dimensions of the perceived employability scale. As showed in Table 4, the hypothesized model provided a better fit to the data than the alternative one-factor model in both samples. These results, together with the reliability estimates (Cronbach’s alpha; Table 1), support the validation of the newly created scale of perceived employability intended for unemployed individuals.

**Table 4:** Confirmatory factorial analysis for employees (*n* = 181) and unemployed (*n* = 237) samples.

Models	χ^2^	df	GFI	RMSEA	TLI	CFI	IFI	Δχ^2^	Δdf
S_1__Model 1D	802.86	104	0.54	0.193	0.49	0.56	0.56		
S_1__Model 4D	321.83	98	0.81	0.112	0.83	0.86	0.86	M_4D_ - M_1D_ = 481.03^∗∗∗^	6
S_2__Model 1D	181.84	20	0.84	0.186	0.80	0.86	0.86		
S_2__Model 2D	174.28	19	0.85	0.186	0.79	0.85	0.86	M_2D_ - M_1D_ = 7.56^∗^	1

*S_*1*_ = employed; S_*2*_ = unemployed; S_*1*__Model 1D = Sample 1, 1 dimension; S_*1*__Model 4D = Sample 1, 4 dimensions; S_*2*__Model 1D = Sample 2, 1 dimension; S_*2*__Model 2D = Sample 2, 2 dimensions. ^∗^*p* < 0.05, ^∗∗∗^*p* < 0.001*.

### MANOVA

Finally, we performed MANOVA in order to analyze differences between sex and gender identity profiles and their interaction in perceived employability in both samples.

#### Sample 1: Employees

The main effect of sex in the four dimensions of perceived employability (RQ1) was not significant (Wilks’ Lambda = 0.956, *p* = 0.10, η^2^= 0.044), probably owing to the relatively small sample size. Analyzed separately, there were significant sex differences in perceived internal quantitative employability [*F*_(1,174)_ = 5.15, *p* = 0.024, η^2^= 0.029] and perceived internal qualitative employability [*F*_(1,174)_ = 6.96, *p* = 0.009, η^2^= 0.038]. Men perceived to be more employable in the internal (quantitative and qualitative) labor market. There were no significant differences in perceived external quantitative employability [*F*_(1,174)_ = 0.014, *p* = 0.906, η^2^= 0.000] and perceived external qualitative employability [*F*_(1,174)_ = 0.003, *p* = 0.0954, η^2^= 0.000].

The main effect of gender identity (RQ2) was not statistically significant (Wilk’s Lamda = 0.917, *p* = 0.24, η^2^= 0.029). Analyzed separately, differences were significant for perceived external qualitative employability [*F*_(3,174)_ = 3.15; *p* = 0.026, η^2^= 0.052]. *Post hoc* analyses performed with Bonferroni showed significant differences between androgynous and undifferentiated gender profiles: the androgynous profile scored significantly higher than the undifferentiated profile. Differences were not significant in perceived external quantitative employability [*F*_(3,174)_ = 1.56, *p* = 0.200, η^2^= 0.026], perceived internal quantitative employability [*F*_(3,174)_ = 1.76, *p* = 0.157, η^2^= 0.029] and perceived internal qualitative employability [*F*_(3,174)_ = 2.00, *p* = 0.115, η^2^= 0.033].

The interaction effect of sex and gender identity to the different dimensions of perceived employability (RQ3) was not significant: Perceived external quantitative employability [*F*_(3,174)_ = 0.874; *p* = 0.456, η^2^= 0.015] and perceived external qualitative employability [*F*_(3,174)_ = 0.519, *p* = 0.670, η^2^= 0.009], perceived internal quantitative employability [*F*_(3,174)_ = 0.653, *p* = 0.582, η^2^= 0.011] and perceived internal qualitative employability [*F*_(3,174)_ = 0.089, *p* = 0.966, η^2^= 0.002].

In concert, our conclusion is that both sex and gender identity relate to perceived employability. Employed men felt more employable, both quantitatively and qualitatively, in the internal labor market than women (RQ1), and employed individuals in the androgynous profile expressed higher qualitative internal employability than individuals in the undifferentiated profile (RQ2). The interaction between sex and gender identity was not significantly related to perceived employability in the sample of employed.

#### Sample 2: Unemployed

The main effect of sex (RQ1) was significant (Wilks’ Lambda = 0.963, *p* = 0.013; η^2^= 0.037), for both perceived external quantitative employability [*F*_(1,229)_ = 5.21, *p* = 0.023; η^2^ = 0.022] and perceived external qualitative employability [*F*_(1,229)_ = 8.48, *p* = 0.004; η^2^ = 0.036]. Women perceived to be more employable than men.

On the contrary, there were no significant gender identity differences on the dimensions of eternal perceived employability (RQ2) (Wilks’ Lambda = 0.959; *p* = 0.148; η^2^= 0.020). When analyzed separately: Perceived external quantitative employability [*F*_(3,229)_ = 1.93, *p* = 0.126; η^2^ = 0.025] and perceived external qualitative employability [*F*_(3,229)_ = 1.57, *p* = 0.198; η^2^ = 0.020].

**FIGURE 1:**
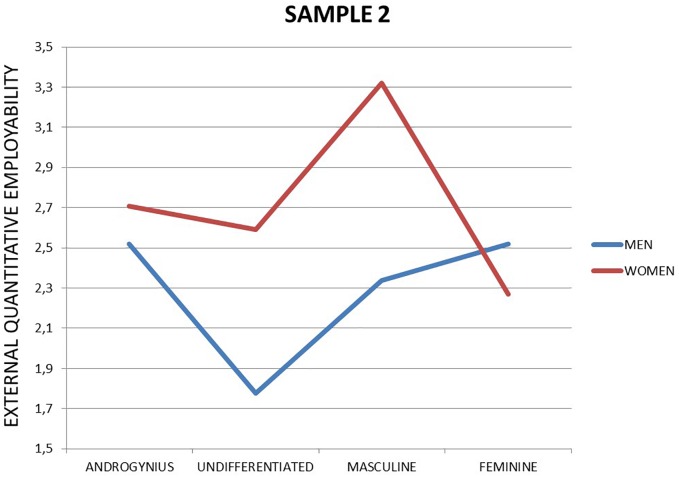
External quantitative employability among unemployed (Sample 2).

**FIGURE 2:**
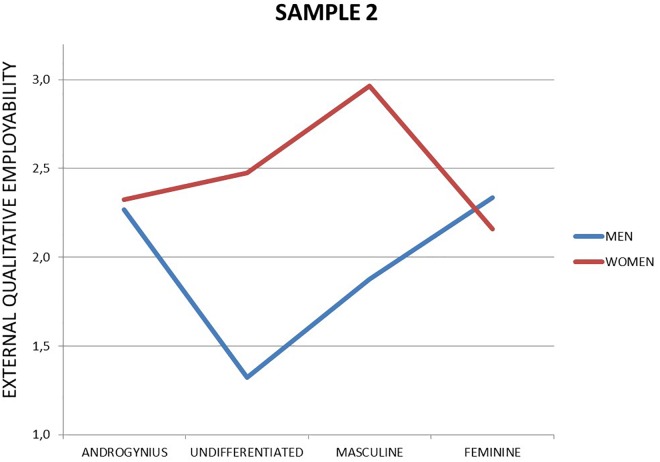
External qualitative employability among unemployed (Sample 2).

Finally, the interaction between sex and gender identity (RQ3) was significant for perceived external qualitative [*F*_(3,229)_ = 4.19, *p* = 0.006; η^2^ = 0.052] but not for perceived external quantitative employability [*F*_(3,229)_ = 2.49, *p* = 0.061; η^2^ = 0.032]. Although the interaction in perceived external quantitative did not meet commonly accepted significance levels, it showed a small but valuable effect size. We therefore detail the interaction in Figures 1, 2.

As the figures show, being feminine among the unemployed relates differently to perceived employability among men and women. Feminine men have the highest score in perceived external quantitative employability, whereas feminine women have the lowest. The highest perceived external qualitative employability scores are for masculine the lowest and worst for undifferentiated men.

Overall, we established that unemployed women felt more employable than men in the external labor market (RQ1). Gender identity did not relate to perceived employability when studied as a main effect (RQ2), yet the interaction with sex was significant in relation to perceived external qualitative employability and meaningful (though not significant) for perceived external quantitative employability (RQ3).

## Discussion

This study provides a gendered perspective on perceived employability among employed and unemployed Spanish youngsters for whom employability has particularly resonance. Spain has a high rate of unemployment among youngsters; hence, providing insight in the potential barriers to employability, both in terms of finding and maintaining employment is a core issue.

The pattern of results was perhaps most remarkable for sex differences (RQ1). *Employed* young men perceived more chances in the internal labor market, both quantitatively and qualitatively, than women, but not in the external labor market. Thus, young men perceive that they have more employment opportunities within their organization than young women. This is in line with the available evidence and discussion regarding sex disparities in employment and promotion in organizations (i.e., Cifre et al., 2015; Quinn and Smith, 2018). It furthermore aligns with the idea that men, women, and organizational policies might routinely engage in practices of “doing gender” that reproduce gender inequality, even if unconsciously (i.e., Schilt, 2006; Huffman et al., 2010). In sum, these differences in perceived internal employability might be rooted in societal, cultural, and organizational barriers that preclude females to have same opportunities as men (i.e., glass ceiling). It is however against the idea often advanced in employability and career studies that individuals themselves carry most responsibility over their career (Forrier et al., 2018). It seems that women still today perceive boundaries to employment opportunities within the organization, possibly due to the perception of stereotyped bias (Heilman, 2012) for women’s career progress. However, we did not identify sex differences regarding external perceived employability. A plausible explanation could be that there is a common feeling that there are few job opportunities out there, so youngsters who have a job are very much focused upon keeping the present job: they may have become risk-averse owing to the recent crisis.

The pattern of results for unemployed was quite different. Unemployed young women perceived to be more employable, both quantitatively and qualitatively, than men. These results agree with those showed by official figures on Spanish employment among youngsters: women more easily find a job, though those jobs are often of lower quality (i.e., part-time jobs). This may seem at odds with women’s perception of “qualitative employability.” It could be that women consider part-time jobs in a positive way, for example in view of facilitating work-home issues. This pattern also seems to suggest that individuals take contextual features into account when appraising their labor market chances. A further explanation is that men are traditionally seen as “bread provider”: when unemployed, men may feel they failed, and this may cause a loss of self-confidence and self-efficacy, and ultimately show lower perceptions of being employable. An related explanation is that men, more than women, are penalized for being unemployed by the environment such as potential future employers, peers and partners, and this may well translate into lower feelings of being employable.

Gender identity was related to perceptions of employability in the employed sample, but not in the unemployed sample (RQ2). Among the employed, androgynous men and women score higher in all dimensions of perceived employability, yet the difference was significant only for perceived external qualitative employability in comparison to the undifferentiated profile. Androgyny refers to an adaptive personality character structure in which masculine and feminine traits are integrated in a person regardless of their sex (Barberá, 1998). The expression of these traits in an integrated or separate way will depend on the situation (Bem, 1977; Baldwin et al., 1986), which make them more adaptable to different situations. Those balanced feminine-masculine traits are supposed to be an advantage when obtaining an external job, as it seems that they show higher levels of emotional intelligence (Gartzia, 2010; Gartzia et al., 2012) than people with a more stereotyped identity. Besides, although not statistically significant, men and women with a masculine gender identity score higher in perceived employability. So, it seems that there is a trend for unemployed masculine men and women to perceive that they have more chances in the labor market than the other profiles, maybe because this market value mostly personality traits associated with masculinity (i.e., agentic traits).

Also interesting is the analysis of the interaction between sex and gender identity (RQ3). Overall, men identify mostly with masculine traits (32.1%) and less with feminine ones (15.8%), while women identify mostly with feminine traits (37.4%) and less with the masculine ones (14.2%). Thus, it seems that although research on gender identity is based on stereotyped gender traits that began on the seventies of twentieth century (i.e., masculine men, feminine women), this profile is still validated among young people 40 years later, at least in Spain. Nevertheless, we can see a different trend if we focus on unemployed vs. employed sample. There were proportionally more feminine men and women in the unemployed compared to the employed sample, and conversely, more masculine women in the employed sample. Again, these results might suggest that masculine traits are still the most searched by organizations, independently of their sex (men-women), so young individuals with more masculine traits and less feminine ones are those that feel more employable. This result, together with the previous one regarding the highest internal perceived employability by employed men, could address to what is called the theory of gendered organizations by Acker’s (1990). This theory proposes that pervasive gender inequities are produced and legitimized through institutionalized policies, communication patterns, organizational bodies, social structures, and divisions of labor, perpetuating disparities in power that explicitly and implicitly advantage men over women. This theory has been recently confirmed even in so seemingly gender neutral organizations such as academia (Conesa Carpintero and González Ramos, 2018; Hanasono et al., 2018).

Although sex by gender interaction is not significant to account for any difference in perceived employability (RQ3) in employees, it was significant among those who are unemployed. First of all, it is worthy to remark that young unemployed women score higher than men in perceived employability, except those with a feminine profile. Besides, it seems that women with high masculine gender identity are those that perceive the highest external quantitative and qualitative employability, even much more than masculine men. Therefore, it seems that “masculine” or more agentic women (as suggested before) feel that they adapt better to the requirements of the labor market, so they feel prepared when searching for a new job. This is especially interesting since the highest score in perceived employability among unemployed men is the lowest score for unemployed women: being feminine (or androgynous in the case of men). It seems that to distinguish from other in the job search, both must adopt a gender identity theoretically incongruent with their sex: men must show feminine traits while women masculine ones. Surprisingly, these results are unexpected regarding the role congruity theory (Eagly and Karau, 2002), which proposes (focusing on female leaders) that perceived incongruity between the female gender role and leadership roles leads to two forms of prejudice (a) perceiving women less favorably than men as potential occupants of leadership roles and (b) evaluating behavior that fulfills the prescriptions of a leader role less favorably when it is enacted by a woman. However, in this case it seems that this role incongruity seems to promote a higher level of perceived employability among unemployed. However, this theory is based on perception of others. In this case, perceived employability is about self-perceptions. So, going a step further, it seems that explained before, youngest might feel that being gender-incongruent might open new options in the labor market, since it can differentiate them from the rest of the competition and give them access to new job niches (i.e., care of others jobs in the case of men). Nevertheless, being undifferentiated or masculine are the worst options for unemployed men perceived employability, as it might seem that they do not accomplish their bread-winner gender role according to the social role theory (Eagly, 1987). Summing up, it seems that showing personality traits incongruent with their sex might be a differential component to increase their perceived employability in a different way to men and women.

### Theoretical and Practical Implications

This study moves employability research forward. It highlights the role of sex, gender identity and their interaction in shaping perceptions of employability across employed and unemployed youngsters. Though the pattern of results is far from straightforward, it is clear that perceived employability is dependent upon sex and gender, in both employed and unemployed youngsters.

Concerning sex, women seem less confident about their chances of maintaining or improving their job within an organization, while men are less confident about their chances to obtain a new job when unemployed. While these findings may appear conflicting, they align with gender roles: women still are disadvantaged in organizational life, yet men are penalized more heavily when unemployed. This aligns with Social Role Theory (Eagly, 1987) which posits that women are expected to perform roles in the private sphere, whereas society expects men continue developing the “bread-winner” role in the public one. So men are more supported to do that and, if not, they are penalized.

Concerning gender identity, the androgynous gender profile in the employed sample (both men and women) scored higher on perceived external qualitative employability, probably because they can more easily adapt to different situation and adaptability is traditionally seen as key to employability (Forrier et al., 2015).

Concerning sex and gender identity interaction, results show that those young unemployed that identified with an incongruent sex–gender identity profile (i.e., masculine women and feminine men) presented the highest levels of perceived employability. These results go a step beyond the role congruity theory (Eagly and Karau, 2002) which proposes that perceived incongruence between the female gender role and leadership roles leads to prejudice that might affect women negatively. In this case, it seems that both unemployed men and women feel that when displaying those incongruent traits, they have more opportunities in the labor market. However, this should not imply that we should encourage the expression of incongruent gender identity. Current research has shown than higher levels of congruence between one’s inner self-concept and outward expression of their identity (action authenticity) may allow one to focus and to enjoy work, and this brings about positive outcomes such as job satisfaction (Martinez et al., 2017).

Overall, our conclusion is that sex and gender identity do play a role in shaping employability perceptions. One practical implication of these results is that employability is *not* all about upskilling and increasing competences, but (at least in part) also structurally determined (see e.g., Forrier et al., 2018). Perceptions of being employable are affected by who you are or how have you been categorized by birth (sex), by the associated roles you are expected to play (gender), and less so by who you feel you are (gender identity). These results are especially dramatic if we consider that we are focusing on young individuals (under 30), who are continuously receiving messages about what to do to be more employable, seeming that employability falls entirely under their responsibility or under their control. However, it seems that is our society itself which has a hard work to do to break stereotypes associated to men and women at work that would mean a step forward to equal access to the labor market.

This results highlight that more structural elements, such as gender roles (as expressed in sex and gender differences), should be feature more prominently in employability studies, and not just as control variables.

### Study Limitations

This study has been performed with young employed and unemployed people in Spain. Therefore, as our sample was younger than 30, we cannot generalize this results to older age groups. Different patterns may emerge within different age groups. So although it was interesting to examine that gender identity still followed gender stereotypes in young generations, future studies should confirm these results including a wider range of age.

Also the study was focused on the Spanish context. Future studies should replicate these findings in a cross-national study, to examine the role played by cultural contexts when predicting perceived employability. Spain has been traditionally a catholic country with a strong patriarchal model. It would be interesting then replicating this study in Northern Europe countries with a more liberal model and where gender equality is almost a reality.

This was an exploratory study, where the relationship between sex, gender identity and perceived employability has been analyzed in a descriptive way. Future longitudinal studies could go a step forward analyzing from a gendered point of view more complex models including antecedents (e.g., self-efficacy/self-esteem or perceived barriers to career) and consequences of perceived employability for careers and occupational health.

Finally, our samples are relatively small, so it would be interesting replicating these results with a larger sample. However, this study includes two samples (employed and unemployed) that allows us to examine sex/gender and gender identities taking into account structural variables such as the employment status.

### Strengths

A particular strength is that we situate this gendered perspective in two samples of employed and unemployed Spanish youngsters, for whom perceptions of employability have particular resonance.

In addition, we provided a broad account on both gender and perceived employability. With respect to gender, we included sex, gender identity, and their interaction. With respect to perceived employability, we included combinations of perceived chances in the internal and external labor market, and perceived overall and qualitative chances.

To perform this study, we have created a new scale for perceived qualitative and quantitative external employability for the unemployed sample, based on the existing scale for perceived external qualitative and qualitative employability. Reliability indices and CFA show that this (Spanish) scale has appropriate psychometric properties to be used in future studies.

Finally, although our study was based on heteronormativism, considering binary only men-women, we included the interaction between sex and gender identity to go beyond the stereotyped assigned men-masculine and women-feminine associations. We hope that this study represents a step forward in understanding the role of sex/gender and gender identities in organizations in particular and in societies in general.

## Conclusion

In conclusion, our results highlight the importance of sex and gender identity for employability. Young people sex (men/women) as well as gender identity they assume (agentic vs. communal traits) will affect their perceived employability, and the way they approach to the labor market. This labor market is still gendered (i.e., employed men perceive that have more chances to get a better job into their organization, unemployed masculine men and women perceive they have more chances in the labor market). The implication is that we might shift the focus and responsibility from the individual to include also other stakeholders (e.g., employers, policy-makers,…). Only being aware of the stereotyped bias performed by organizations and assumed by individuals, we will be able to contribute to build more equal societies. From a feminist compromise, those changes should be promoted from all different social fields, including science. Hope this article contributes to it.

## Ethics Statement

All participants provided written informed consents before to complete the survey, in accordance with the Declaration of Helsinki, and researchers guaranteed the anonymity of data. The protocol was approved by the ethics committee of Universitat Jaume I.

## Author Contributions

All authors contributed to this work. EC designed the study, collected the data, wrote the first version of the introduction and the discussion of the manuscript, and was the supervisor of the research team, so works as first author. MV and IS-C performed data analysis and wrote the first version of the “Materials and Methods” and “Results” section. NdC reviewed and included the employability literature in the “Introduction” and “Discussion” section. All of them repeatedly revised the manuscript. All authors approved the final version of the manuscript to be published.

## Conflict of Interest Statement

The authors declare that the research was conducted in the absence of any commercial or financial relationships that could be construed as a potential conflict of interest.

## Footnotes

1 Definitions according to David (2009): Gender and sex: The condition of being female, male, neuter, or androgynous. In recent times there has been a differentiation between sex and gender in describing human beings and to a lesser extent other animals such that sex refers to the biological aspects of femaleness and maleness while gender refers to the cultural, social, and psychological aspects of being defined as female or male. Gender identity: Is the identification of one-self as female or male; that means that gender identity is a cognitive process distinct from gender role behaviors. Gender role: It is a learned set of behaviors associated with women or men. These behaviors are so strongly associated with each sex that the set of behaviors comes to define masculinity or feminity in any given culture. The underlying basis for gender roles is biological sex differences, but most authorities agree that gender role behaviors are learned.
